# Determinants of HIV infection among infants born to HIV positive women receiving option B + prevention of mother to child transmission of HIV in Tigray, north Ethiopia: a case control study

**DOI:** 10.1186/s12981-025-00755-3

**Published:** 2025-05-30

**Authors:** Haftay Gebremedhin, Fre Gebremeskel, Gebremedhin Gebreegziabiher, Abadi Hailay Atsbaha, Gebretekle Gebremichael Hailesilase

**Affiliations:** 1 College of Medicine and Health Sciences, Department of Public Health, Adigrat University, P.O. Box: 50, Adigrat, Tigray Ethiopia; 2 College of Medicine and Health Sciences, Department of Pharmacy, Adigrat University, P.O. Box: 50, Adigrat, Tigray Ethiopia

**Keywords:** Ethiopia, HIV, Infants, Option B + PMTCT, Tigray

## Abstract

**Background:**

The option B^+^ prevention of mother to child transmission of human immunodeficiency virus is the lifelong provision of antiretroviral therapy for all human immunodeficiency virus positive pregnant and breastfeeding women regardless of immune status. In Ethiopia, the overall mother-to-child transmission rate of human immunodeficiency virus was 15.9%. This study assessed determinants of human immunodeficiency virus infection among infants born to human immunodeficiency virus positive women on option B + prevention of mother to child transmission of human immunodeficiency virus in Tigray, north Ethiopia.

**Methods:**

Unmatched case-control study was conducted in Tigray region from October 2023 to April 2024. A total of 43 cases and 129 controls were selected using simple random sampling technique. Multivariable logistic regression analysis was fitted to identify the factors associated with mother to child transmission of human immunodeficiency virus at *P* < 0.05. Multicolinearity was checked among predictor variables using Variance Inflation Factor and Tolerance test. Furthermore, the goodness of fit of the logistic model was tested using Hosmer-Lemshow test.

**Results:**

This study showed that rural residence (Adjusted Odds ratio: 33.3, 95% CI: 1.02–87.05), World Health Organization disease stage III (Adjusted Odds ratio: 57.4, CI: 9.25– 297.54) and IV (Adjusted Odds ratio: 78.9, CI: 12.64–345.62) during initiation of antiretroviral therapy and a child with mouth ulcer during exclusive breastfeeding (Adjusted Odds ratio: 65, IC: 6.39–456.23) were the factors significantly associated with mother to child transmission of human immunodeficiency virus. Besides, mothers’ educational status (Adjusted Odds ratio: 0.2, CI: 0.04, 0.35), late time of antiretroviral therapy initiation after human immunodeficiency virus diagnosis (Adjusted Odds ratio: 0.14, CI: 0.02–0.18) and absence of human immunodeficiency virus exposed infant follow up visit (Adjusted Odds ratio: 0.04, IC: 0.005–0.09) had significant association with the mother to child transmission of human immunodeficiency virus.

**Conclusion:**

The determinant factors significantly associated with mother to child transmission of human immunodeficiency virus were identified. Health care providers should strengthen option B + prevention mother to child transmission of human immunodeficiency virus services to reduce the mother to child transmission of human immunodeficiency virus.

## Introduction

The option B + prevention of mother to child transmission (PMTCT) of human immunodeficiency virus (HIV) is the lifelong provision of antiretroviral therapy (ART) for all HIV positive pregnant and breastfeeding women regardless of immune status [1]. The option B + PMTCT interventions such as HIV testing and counseling (test and treat principle), maternal fixed dose combination ART, infant nevirapine prophylaxis, early infant HIV diagnosis, and maternal-infant follow-up visit until breastfeeding cessation have become widely available. Based on these conditions, mother to child transmission (MTCT) of HIV throughout the perinatal period has been reduced from a baseline 25–45% over a decade ago to less than 5%. MTCT of HIV is a vertical transmission of HIV from an HIV contracted mother to her child [2, 3].

Globally, in 2008, 500,000 children worldwide were newly contracted by HIV [4]. A 60% declined in new HIV diagnosis was observed among children in the Global Plan Priority countries between 2009 and by the end of 2015 [5]. More than 90% of vertical transmission of HIV infections had occurred in low and middle-income countries. The likelihood of HIV transmission without treatment from mother to child is 15–45% [6]. Botswana, the only non-breastfeeding Global Plan Priority country, had a transmission rate of 2.6%, just above the threshold of 2.0% [5]. Other effective PMTCT interventions and ART could reduce the risk to the level of below 5% [7, 8]. The World Health Organization (WHO) recommends that HIV-positive women should exclusively breastfeed their infants until six months and continues breastfeeding until twelve months [9]. At six weeks of age, all infants born to HIV-positive mothers should be given early infant diagnosis using deoxyribonucleic acid polymerase chain reaction (DNA/PCR) test. Another HIV test should be done at 18 months and/or when breastfeeding ends to provide the final infant diagnosis [10].

According to the 2003 United Nations Children’s Fund (UNICEF) report, in a half million women who attended clinics, only 71% received counseling; of those who were counseled, only 70% took HIV test; among those tested HIV positive, only 49% received preventive drugs [11]. Among women who visited clinics only once during pregnancy, nearly two-thirds of them used to give birth in the absence of skilled health workers [12]. Thus, the above problems had contributed to increase the number of HIV contracted infants in Sub-Saharan Africa countries. According to the United Nations acquired immunodeficiency syndrome (UNAIDS) 2016 report, the number of new HIV infections among children declined to less than 20,000 by 2020 [13].

The UNAIDS 2017 report showed that the overall MTCT rate of HIV was highest in Indonesia (26.6%) followed by Angola (21%) and Ghana (17.7%). In Ethiopia, the 6 weeks, post 6-week and overall MTCT rate of HIV were 7.6%, 8.4% and 15.9%, respectively [14]. Previous study indicated that the risk of in utero HIV transmission is greater among women who have their primary HIV diagnosis during pregnancy compared with women who were contracted prior to conception [15]. With the rising prevalence rate of HIV, about 12,000 new diagnosis occurred in Ethiopia in 2020, most of which are due to MTCT accounting for 90% of new infections [2]. Moreover, Ethiopia had an estimated adult prevalence of 1.5% [16]. On the other hand, reports in 2011 showed that in Ethiopia, under five and neonatal mortality rates were decreased from 123 to 88 and from 39 to 37 per 1,000 live births, respectively [17]. In June 2014, 98.1% of expectant mothers have accessed antenatal care (ANC) services at least once in Ethiopia. The proportion of practicing mixed feeding within the first six months of life among HIV positive infants born from HIV positive mother was 31.1% in the country [18]. In the year 2013–2016, the national and regional MTCT rate of HIV in Ethiopia was 11% and 12.2%, respectively [19].

The effectiveness of option B + PMTCT might be different across different settings attributed to many factors such as differences in the availability of resources for HIV positive mothers follow up, national PMTCT policy, patient socio-demographics status, healthcare providers commitments and so on. In a study done in elsewhere in Ethiopia, HIV transmission rate was highest among those who took either no prophylaxis or single dose nevirapine [20]. Another study showed that factors such as maternal malnutrition, unplanned pregnancy, home delivery and mixed feeding of child during first six months of life were determinants of MTCT of HIV [21]. Although the PMTCT strategy has been practiced in Ethiopia, the rate of MTCT of HIV among infants born to HIV positive mothers was reported to be 15% in 2020 in the country [7], which is far from the targeted zero by 2020 set by the UNAIDS [2].

To the best of our knowledge, there is no study conducted on determinants of HIV transmission among infants born to HIV positive women receiving option B + PMTCT of HIV in Tigray, north Ethiopia. Therefore, this case control study was aimed to clearly identify determinants of HIV transmission among infants born to HIV positive mothers receiving option B + PMTCT of HIV. Findings of this study will serve as an input to policy makers, health professionals working on PMTCT, experts and managers in health facilities and researchers who are interested in this area.

### What is already known

- Ethiopia is highly affected by HIV having an estimated adult prevalence of 1.5%.
- Although the PMTCT has been practiced in Ethiopia, the rate of MTCT of HIV among infants born to HIV positive mothers remains far from the targeted zero set by the UNAIDS.
- The national and regional MTCT rate of HIV in Ethiopia is high, which was 11% and 12.2% respectively, in the year 2013–2016.

### What is unknown

- The determinants of HIV transmission among infants born to HIV positive women receiving option B + PMTCT of HIV in Ethiopia in general and in Tigray (north Ethiopia) in particular are poorly studied.

## Methods

### Study area and period

The study was conducted in Tigray regional state, north Ethiopia. Tigray regional state is located in northern part of Ethiopia bordered by Eritrea to the north, Sudan to the west, Afar region of Ethiopia to the east, and Amhara region of Ethiopia to the south. According to the projected census of 2007, the Tigray region had a total population of 4,806,843, of whom 2,441,158 (50.8%) were women; and 1,019,176 (21.2%) were urban inhabitants [22]. There are fifteen government general hospitals, 3 private hospitals, and 256 health centers in the Tigray region. The study was conducted from October 2023 to April 2024.

### Study design

Unmatched case control study design was conducted among HIV positive mothers who were on option B + PMTCT services in Tigray regional state with their children who had confirmed HIV test results at 6 weeks of age.

### Study population

A case was defined as HIV-positive mother who had been on option B + PMTCT services with her child tested confirmed HIV positive at 6 weeks of age. On the other hand, a control was defined as HIV positive mother who had been on option B + PMTCT services with her child tested definitive HIV negative at 6 weeks of age. The HIV status of the mother and child was obtained from their records. Children accompanied by someone else other than their mothers or whose mothers were transferred out to facilities outside of the study area were excluded from the study.

### Sample size determination and sampling technique

#### Sample size determination

Sample size was calculated considering the following assumptions; confidence interval of 95%, the power level = 80%, r = the ratio of cases to controls 1 to 3, the calculated Odds ratio of 6.4, proportion of practiced mixed feeding within the first six months of life among cases (HIV positive infants) 31.1% and proportion of practiced mixed feeding within the first six months of life among controls (HIV negative infants) 6.6% [18]. The sample size needed was 28 cases and 84 controls. Then, with the consideration of design effect 1.5 and none response rate of 5%, a total of 172 study participants (43 cases and 129 controls) were finally included in the study.

#### Sampling technique

From a total of fifteen general hospitals providing option B + PMTCT services in Tigray regional state, six general hospitals were selected using simple random sampling technique found in Maychew, Mekelle, Adigrat, Adwa, Abi Adi and Aksum. All the hospitals sent a dried blood sample of HIV exposed infants to Tigray regional laboratory diagnosis center for DNA/PCR-HIV test and the confirmed HIV status of the infant was return back to the health facilities within a month. All health facilities were expected to report the confirmed HIV-positive infant to Tigray Regional Health Bureau. Among all health facilities reporting to Tigray Regional Health Bureau, at least one HIV-positive child born from an HIV-positive mother on option B + PMTCT services was selected. Based on the total number of infants’ HIV status (cases and controls), the calculated sample size was proportionally allocated to the selected hospitals. Accordingly, a total of 43 cases and 129 controls were selected using simple random sampling technique.

### Study variables

#### Outcome variable

Infant HIV status at 6 weeks HIV test. If the infant is HIV positive, it is defined as case. If the infant is HIV negative, it is defined as control.

#### Predictor variables

Socio-demographic and economic status, mothers related, infants related, drug adherence, and previous personal habits of mother during treatment were the independent variables.

### Data collection tools and procedures

The data collection tool was developed by reviewing previously published articles [19–24]. Data were collected using a structured questionnaire from the mothers and infants’ cohort registration book review. The study variables of interest were prepared based on the contents of health facility HIV/AIDS care records in touch of or directly addressing option B + PMTCT and registrations of ART to an infant and their mothers. The document review administered questionnaire had socio demographic and economic conditions, infant HIV status, health and health care related factors, obstetric related variables and mothers’ behavior. The entire mother paired cohort registration book, HIV exposed infant card and health management and information system were available during data collection period.

### Data quality control

The principal investigator trained the data collectors and supervisors for two consecutive days on instruction for the method, ethical procedure, general information on option B + PMTCT and objective of the study. Twelve BSc nurses working on PMTCT and ART clinic, and four masters of public health students were assigned as data collectors and supervisors. The questionnaire was translated into local language Tigrigna and back translated into English by translators who were blind to the original questionnaire. That means, two BSc nurses were assigned in each health facility as data collectors and two supervisors were assigned in three health facilities. The questionnaire was pre-tested on 5% of study population in non-selected health facilities. After data collection, data were stored in a secured place to maintain confidentiality and backup of data was stored in different areas to prevent loss of the data.

### Data management and analysis

The collected data were coded, entered, cleaned and analyzed using Statistical Package for Social Sciences version 20. Descriptive statistics was used to describe HIV positive and negative infants born from HIV positive mothers. To select the candidate variables, Crude Odds Ratios and their 95% confidence of interval with the *P* < 0.2 were estimated using univariable logistic regression analysis to include in multivariable logistic regression model. After adjusted for confounders, Odds Ratio with 95% confidence interval and p-value < 0.05 was considered to declare statistically significant. Before inclusion of predictors to the final logistic regression model, the multicolinearity was checked among predictor variables using variance inflation factor [ (VIF), < 10] and minimum tolerance test (Tolerance tests > 0.1). The goodness of fit of the final logistic model was tested using the Hosmer-Lemeshow test.

### Operational definitions

#### Mothers on option B + PMTCT

Mothers who had taken antiretroviral for prevention of MTCT of HIV either during pregnancy or childbirth/delivery or during breastfeeding [25].

#### Definitive HIV test results

HIV test result of a child identified with dried blood sample of DNA/PCR test at 6 weeks of age [25].

#### Case (HIV positive infants)

Is a child who tested at least one HIV positive result using DNA/PCR test at 6 weeks of age.

#### Control (HIV negative infants)

Is a child who tested at least one HIV negative result using DNA/PCR test at 6 weeks of age.

#### ART adherence

Measured based on number of missed doses within 60 days. Three or less doses, four to nine doses and more than nine doses were rated as good, fair and poor respectively [25].

#### Infant mouth ulcer

This was clinically diagnosis taken from mother/infant registration book.

## Results

### Socio-demographic characteristics of study participants

A total of 172 respondents (43 cases and 129 controls) were included in the study. Most of the mothers of the cases (72.1%) were in the age range of 20–24 years while the remaining (27.9%) were in the age range of 25–29 years. Around 31(72.1%) of cases and 18(14%) of controls were rural residents. Regarding educational status,, three-quarters (74.4%) of mothers could not read and write among the cases (Table 1).

**Table 1:** Socio-demographic and economic characteristics of respondents receiving option B + PMTCT services, Tigray region, North Ethiopia, October 2023 to April 2024

Variables		Status of the parents of the infants			
		Cases		Controls	
		Frequency (n)	Percent	Frequency (n)	Percent
Age of mothers (Years)	20–24	31	72.1%	27	20.9%
	25–29	12	27.9%	85	65.9%
	≥30	0	0.0%	17	13.2%
Place of residence	Urban	12	27.9%	111	86.0%
	Rural	31	72.1%	18	14.0%
Religion	Orthodox	18	41.9%	112	86.8%
	Muslim	21	48.8%	17	13.2%
	Protestant	4	9.3%	0	0.0%
Ethnicity	Tigray	19	44.2%	107	82.9%
	Afar	20	46.5%	16	12.4%
	Amara	4	9.3%	6	4.7%
Educational status of mother	Cannot read and write	32	74.4%	37	28.7%
	Can read and write(informal)	7	16.3%	3	2.3%
	Read and write (Formal education)	4	9.3%	89	67.0%
Educational status of father	Cannot read and write	13	30.2%	43	33.3%
	Can read and write (informal)	11	25.6%	14	10.9%
	Read and write (formal education)	19	44.2%	72	55.8%
Occupational status of mother	Government employee	1	2.3%	8	6.2%
	Private business	2	4.7%	77	59.7%
	Private sector employee	4	9.3%	1	0.8%
	House wife	36	83.7%	43	33.3%
Occupational status of father	Government employee	4	9.3%	38	29.5%
	Private business	37	86.0%	72	55.8%
	Private sector employee	2	4.7%	19	14.7%
House hold monthly income (Ethiopian Birr)	< 1000	31	72.1%	77	59.7%
	1000–3000	2	4.7%	14	10.9%
	3000–5000	2	4.7%	22	17.1%
	> 5000	0	0.0%	12	9.3%
	Have no monthly income	8	18.6%	4	3.1%
Source of information on PMTCT	Radio	22	51.2%	106	82.2%
	Television	15	34.9%	5	3.9%
	Health worker	6	13.9%	18	13.9%

### HIV positive mothers receiving option B + PMTCT

Concerning mothers’ HIV status, 23 (53.5%) of cases and 63 (48.8%) of controls were diagnosed their HIV status during pregnancy; followed by 17 (39.5%) of cases and 49 (38%) of controls during provider initiated HIV testing and counseling (PIHTC) prior to pregnancy; 2 (4.7%) of cases and 15(11.6%) of controls during postnatal period; and 1 (2.3%) of cases and 2 (1.6%) of controls during labor and delivery. Regarding their WHO disease staging, 3 (7.0%) of cases and 74 (57.4%) of controls were in WHO stage (I) Significant number 12 (27.9%%) of cases and 47 (36.4%) of controls were in WHO stage (II) Majority 20 (80%) of cases and 5 (20%) of controls were in WHO stage III followed by 8 (18.6%) of cases and 3 (2.3%) of controls in WHO stage IV (Table 1).

Regarding mothers’ ART initiation, 27 (62.8%) of cases and 19 (14.7%) of controls had started their ART after 7 days of their HIV diagnosis. Concerning their ART regimens, 29 (67.4%) of cases and 112 (86.8%) of controls started TDF-3TC-EFV (1e) followed by AZT-3TC-NVP (1c) with 2 (4.7%) of cases and 15 (11.6%) of controls. On the other side, majority 38 (88.4%) of cases and 88 (68.2%) of controls did not take cotrimoxazole prophylaxis therapy (Table 1).

### Infants born to HIV positive women receiving option B + PMTCT services

Among all the infants, 29 (67.4%) of cases and 91 (70.5%) of controls were female in sex. Eighteen (41.9%) of cases and 63 (48.9%) of controls were in the age range of 6–10 weeks followed by 13 (30.2%) of cases and 24 (18.6%) of controls were greater than or equal to 16 weeks of age and the remaining of 12 (27.9%) cases and 42 (32.6%) of controls were also in the age range of 11–15 weeks. On the other hand, all the infants were diagnosed their HIV status by the DNA/PCR test at six weeks of age (Table 1).

Negligible number (3; 2.3%) of cases and almost all 129 (97.7%) of controls had taken nevirapine prophylaxis immediately after birth. On the other side, around half (47.5%) of cases and 89.1% of controls had taken nevirapine prophylaxis for 6 weeks duration. Over one-third (37.5%) of cases and almost one-out of ten (10.9%) controls had also taken nevirapine prophylaxis for 12 weeks duration. Only nearly two out of ten (20.9%) cases and all (100.0%) of controls had taken cotrimoxazole prophylaxis therapy. Around 86.0% of cases and all (100.0%) of controls were on exclusive breast feeding during the first six months of life. The remained cases (14.0%) were practiced mixed feeding. Concerning complementary feeding practice, 34 (79.0%) of cases and 29 (22.5%) of controls were started before 6 months of age; 3 (7.0%) of cases and 98 (76.0%) of controls were started at 6 months of age; and 6 (14.0%) of cases and 2 (1.5%) of controls started after six months of age (Table 1).

### Health and health care related factors

Concerning mothers’ gravidity, 17 (39.5%) of cases and 12 (9.3%) of controls had history of greater than five gravidities. Around half (48.8) of cases and very small (1.6%) proportion of controls did not attend ANC visit. More than one-third (37.2%) of cases and only one (0.8%) of controls had developed mouth ulcer during exclusive breast-feeding. The level of drug adherence among the cases and controls were 43.4% and 56.6%, respectively. Thirty-six (83.7%) of cases and 86 (66.7%) of controls did not take their anti-retro viral drugs medication immediately after HIV diagnosis. A great majority (97.7%) of cases and less than half (43.4%) of controls had reported missed dose of their anti-retro viral drugs. More than two third (69.8%) of cases and around one-third (34.9%) mothers of the controls drunk alcohol while they were on their anti-retro viral drugs. About 95.8% of cases and small proportion of controls (4.2%) had no HIV exposed infant follow up visit (Table 1).

### Factors significantly associated with HIV transmission of infants

As shown in Table 2, the multivariable logistic regression analysis showed that rural dwelling HIV positive mothers had significantly higher odds to have HIV positive infants compared to urban dwelling HIV positive mothers though the estimate seems imprecise [Adjusted Odds ratio (AOR): 33.3, CI: 1.02–87.05]. Those HIV positive mothers in advanced WHO disease stage III (AOR: 57.4, CI: 9.25–297.54) and IV (AOR: 78.9, CI: 12.64–345.62) during initiation of ART were 57.4 and 78.9 times more likely to have HIV positive infants than those in WHO stage I, respectively. Besides, those HIV positive mothers who had a child with mouth ulcer during exclusive breast feeding were 65 times more likely to have HIV positive infants than a child with no mouth ulcer (AOR: 65, CI: 6.39–456.23). However, those HIV positive mothers who can read and write (formal education) were 80% less likely to have HIV positive infants than those who cannot read and write (AOR: 0.2, CI: 0.04–0.35). Moreover, those HIV positive mothers who took their ART medication after HIV diagnosis within 7 days were 86% less likely to have HIV positive infants than those who took after 7 days of their HIV diagnosis (AOR: 0.14, CI: 0.02–0.18). Those HIV positive mothers who had an HIV exposed infant follow up visit were 96% less likely to get HIV positive infants than those who had no HIV exposed infant follow up visit (AOR: 0.04, CI: 0.005–0.09) (Table 2).

**Table 2:** Factors significantly associated with HIV positivity infants among women receiving option B + PMTCT services, Tigray region, North Ethiopia, October 2023 to April 2024

Variables		Status of the child (parents)				COR (95%CI)	AOR (95%CI)
		Cases		Controls			
		N	%	N	%		
Place of Residence	Urban	12	27.9%	111	86.0%	1	1
	Rural	31	72.1%	18	14.0%	16.7(7.14, 33.3) *	**33.3 (1.02**, 87.05**)**
Educational status of mother:	Cannot read and write	32	74.4%	37	28.7%	1	1
	Can read and write (informal)	7	16.3%	3	2.3%	0.37(0.09, 1.56)	0.24(0.04,1.75)
	Read and write (formal education)	4	9.3%	89	69.0%	0.10(0.02, 0.16) *	**0.20(0.04**,0.35) **
Time of initiation of ART after HIV diagnosis	Within 7 days	16	37.2%	110	85.3%	0.10(0.05, 0.22) *	**0.14(0.02**,**0.18)**
	After 7 days	27	62.8%	19	14.7%	1	1
WHO disease stage during initiation of ART	WHO stage 1	3	7.0%	74	57.4%	1	1
	WHO stage 2	12	27.9%	47	36.4%	6.3(1.69,23.50) *	7.9(0.12,45.6)
	WHO stage 3	8	18.6%	3	2.3%	65.8(11.33,381.84) *	**57.4(9.25**,297.54) **
	WHO stage 4	20	46.5%	5	3.9%	98.7(21.71,448.51) *	**78.9(12.64**,345.62) **
Duration of EBF	Till 6 months of age	3	7.0%	121	93.8%	0.01(0.001,0.02) *	0.02(0.006,76.45)
	> 6 months age	40	93.0%	8	6.2%	1	1
Antenatal care visit	Yes	39	90.7%	128	99.2%	0.08(0.06, 0.29) *	0.59(0.76, 1.17)
	No	4	9.3%	1	0.8%	1	1
HIV-exposed infants with Follow up visit	Yes	20	46.5%	128	99.2%	0.01(0.001,0.05) *	**0.04(0.005**,0.09) **
	No	23	53.5%	1	0.8%	1	1
Post-natal visit to any health institution	Yes	14	32.6%	106	82.2%	0.11(0.05, 0.23) *	0.02(0.001, 87.398)
	No	29	67.4%	23	17.8%	1	1
Mouth ulcer during EBF	Yes	16	37.2%	1	0.8%	75.9(9.64, 596.61)	**65(6.39**,456.23) **
	No	27	62.8%	128	99.2%	1	1

ART = Antiretroviral therapy; EBF = Exclusive breast-feeding; 95% CI = 95% Confidence interval; COR = Crude Odds ratio; AOR = Adjusted Odds ratio; * = Statistically significant variables

## Discussions

This study tried to identify the possible determinants of HIV transmissions among infants born to HIV positive mothers receiving option B + PMTCT of HIV. Accordingly, the study revealed that HIV-positive mothers from rural residences, advanced WHO disease stage, having a child with mouth ulcer during exclusive breastfeeding, lack of formal education among mothers, delayed ART initiation after HIV diagnosis, and absence of HIV-exposed infant follow-up visit were the identified as factors.

In this study, a negligible number of cases (2.3%) and almost all of controls (97.7%) had taken nevirapine prophylaxis immediately after birth. This finding was almost in line with a study conducted in northwest Ethiopia [23] and Amhara region, Ethiopia [24]. This might be due to low awareness creation to HIV positive mothers on the advantage of ANC follow-up and attending institutional delivery by skilled birth attendants. In this study, only two out of ten (20.9%) cases and all (100.0%) of controls had taken cotrimoxazole prophylaxis therapy. This finding was almost similar to a study conducted in Hawassa, Ethiopia [20]. This might indicate that a lack of prophylaxis increases the chance of the transmission of HIV from mothers to their newborn infants. In our study, a great majority of cases (97.7%) and less than half (43.4%) of controls had reported missed doses of their anti-retroviral medications and the level of drug adherence among the cases and controls was 43.4% and 56.6%, respectively. This finding was similar to a study conducted in Addis Ababa, Ethiopia [21]. The high adherence observed in the controls might be explained by the reduction in viral load caused by good ART adherence.

In this study, rural-dwelling HIV-positive mothers were significantly associated with MTCT of HIV as compared to urban-dwelling HIV-positive mothers. This finding was similar to a study conducted in northwest Ethiopia [23]. This might be due to a lack of access to the nearby health facility for option B + PMTCT services and media as compared to the counterparts. This study revealed that mothers in advanced WHO clinical stages III & IV at enrollment to option B + PMTCT services significantly increased MTCT of HIV as compared to mothers in WHO clinical stage I. These findings were also similar to a study done in Jimma, Ethiopia [26], Woliso, Ethiopia [27], and Amhara, Ethiopia [28]. This might be due to the advanced stage of HIV indicating more opportunistic infections leading to high viral load and immunodeficiency which increases the risk of HIV transmission to their child. In this study, a child with mouth ulcer during exclusive breastfeeding was the determinant factor for the transmission of HIV from HIV HIV-positive mother to the child. This is because of mouth ulcer in infants increases the risk of HIV transmission in the infant during exclusive breastfeeding as the HIV can easily enter the body through the damaged tissue even when mother’s breast milk has low viral load.

This study revealed that the educational status of HIV-positive mothers who can read and write (formal education) were less likely to have HIV-positive infants than those who cannot read and write. This study was almost similar to a study conducted in south Ethiopia [29]. This may be attributed by the fact that those mothers attending a formal education can easily understand the importance of attending option B + PMTCT services as compared to their counterparts. Besides, the HIV-positive mothers with formal education are more likely to have better infancy care compared to those HIV-positive mothers specially in terms of feeding habits. In our study, those HIV-positive mothers who had an HIV-exposed infant follow-up visit were less likely to get HIV-positive infants than those who had no HIV-exposed infant follow-up visit. This finding was similar to a study conducted in northwest Ethiopia [23]. This might be due to access to counseling and treatment services of option B + PMTCT. In our study, HIV-positive mothers who took their ART medication within 7 days of their HIV diagnosis were less likely to transmit HIV to their infants than those who took it after 7 days of diagnosis. The current study was similar to a study conducted in northeast Ethiopia [30]. This is because of early starting of ART can protect them from immune-compromisation. However; our findings needs careful interpretation as there are imprecision estimates indicated by large AORs and wide confidence intervals, which might be due to issues such as small sample sizes.

All infants diagnosed with their HIV status by the DNA/PCR test at six weeks of age were included in the study. The reasons to use early infants diagnosed at six weeks of age were to see the effective intervention of maternal fixed-dose combination antiretroviral therapy, infant nevirapine prophylaxis, early infant HIV diagnosis and maternal-infant follow-up visits until breastfeeding cessation has become widely available before transferred from PMTCT clinic to ART clinic [31, 32].

### Limitations

As this research was conducted using secondary data from mothers’ and their infants’ registration books, there were gaps in getting some of the data for capturing. The relatively small sample size might also affect the power of the test. Nevertheless, the findings of this study would be valuable evidence to evaluate option B + PMTCT program effectiveness and provide a foundation for future intervention.

## Conclusion

The present study identified some of the determinants of HIV transmissions among infants born to HIV positive mothers receiving option B + PMTCT of HIV in the assessed general hospitals. Accordingly, HIV positive women from rural residence, advanced WHO disease stage and having a child with mouth ulcer during exclusive breast feeding and absence of mothers’ educational status were the factors significantly associated with MTCT. Moreover, delayed ART initiation after HIV diagnosis and absence of HIV exposed infant follow up visit were the other factors, which were significantly associated with MTCT. Therefore, findings of this study will provide valuable information for policy makers to scale-up in the PMTCT program. **Recommendations**.

Based on our findings, we forward the following recommendations to stake holders including the health professionals in these hospitals, Tigray regional laboratory diagnosis center for HIV test and Tigray Regional Health Bureau. Public health efforts must be strengthened on preventive strategies to reduce incidence of MTCT of HIV giving special attention to the following issues:

- Improve early ART initiation of ART after HIV diagnosis to prevent MTCT of HIV.
- HIV positive mothers with advanced WHO clinical stages should be given a special attention to reduce the risk of MTCT of HIV.
- Promote awareness or educational status of HIV positive mother.
- Assist to the HIV positive mother of rural residents in terms of transportation to health facilities and awareness creation about PMTCT of HIV.
- Special emphasis should be given to a child with mouth ulcer during exclusive breast feeding as they are at higher risk of MTCT of HIV.
- Further studies should be conducted on option B + PMTCT services and determinants of HIV transmissions among infants born to HIV positive mothers in a broader study areas.

## Data Availability

The datasets used and/or analyzed during this study are available from the corresponding author upon reasonable request.

## Abbreviations and acronyms

AIDS: Acquired Immunodeficiency Syndrome
AOR: Adjusted Odds Ratio
ART: Anti-Retroviral Therapy
DNA/PCR: Deoxyribonucleic Acid Polymerase Chain Reaction
HIV: Human Immunodeficiency Virus
HTC: HIV Testing and Counseling
MTCT: Mother to Child Transmission of HIV
PMTCT: prevention of Mother to Child Transmission of HIV
